# Self-compassion and mindfulness in relation to mind wandering: indirect associations mediated by negative affect

**DOI:** 10.3389/fpsyg.2025.1687442

**Published:** 2025-12-11

**Authors:** Ayumi Umeda, Tomu Ohtsuki

**Affiliations:** 1 College of Human Sciences, Kinjo Gakuin University, Nagoya, Japan; 2 Faculty of Human Sciences, Waseda University, Tokorozawa, Japan

**Keywords:** mind wandering, self-compassion, mindfulness, negative affect, positive affect, multiple mediation analysis

## Abstract

Previous studies using mindfulness training interventions have shown that such training can reduce mind wandering, and this has often been attributed to improvements in executive functions. Instead of focusing on executive functions, the present cross-sectional study examined how affect is involved in the associations of self-compassion and dispositional mindfulness with mind wandering. We used multiple mediation analyses to investigate how self-compassion and dispositional mindfulness are related to mind wandering through positive and negative affect. A web-based survey was conducted with 168 Japanese university and graduate students (105 women; mean age = 21.3 years, SD = 2.9). Self-compassion and dispositional mindfulness were both negatively associated with negative affect and mind wandering. Multiple mediation analyses indicated that negative affect, but not positive affect, statistically mediated the associations of self-compassion and dispositional mindfulness with mind wandering. Descriptively, the indirect association via negative affect appeared somewhat larger for self-compassion than for dispositional mindfulness, although we did not formally test the difference between these indirect paths. These preliminary correlational findings suggest that higher self-compassion and higher dispositional mindfulness may be related to less mind wandering, partly through lower negative affect. In addition, the present results provide preliminary groundwork for future intervention studies that directly compare the effects of self-compassion training and mindfulness training on mind wandering via reductions in negative affect.

## 1 Introduction

Mind wandering is a phenomenon in which attention shifts from the task or activity that is currently being performed to unrelated internal information (Smallwood and Schooler, 2006). It has been shown to occur at least 30–46.9% of the time when it is present (Killingsworth and Gilbert, 2010), making it a common and everyday phenomenon. While mind wandering is a commonly experienced phenomenon, its susceptibility is associated with various psychological issues such as depression, anxiety, rumination, high stress perception, low self-esteem, and life satisfaction (Chaieb et al., 2022; Mrazek et al., 2013). Smallwood et al. (2009) demonstrated that inducing negative affect increases mind wandering. Additionally, in a study using the experience sampling method of Killingsworth and Gilbert (2010), mind wandering was shown to be a cause of negative affect. Subsequent studies have consistently shown through various experimental procedures that mind wandering is associated with negative affect (e.g., Deng et al., 2014; Poerio et al., 2013; Stawarczyk et al., 2013). Based on these findings, it can be assumed that mind wandering and negative affect mutually reinforce each other and that there is a negative spiral between mind wandering and negative affect. Furthermore, mind wandering has been associated with schizophrenia (Shin et al., 2015), depressive disorder (Hoffmann et al., 2016; Welhaf et al., 2024), obsessive-compulsive disorder (Seli et al., 2017b), and attention deficit/hyperactivity disorder (Mowlem et al., 2019). Therefore, mind wandering can be considered a diagnosis-transcending phenomenon that may contribute to the onset or maintenance of various mental disorders.

Within the field of clinical psychology, research has primarily examined rumination and worry as cognitive processes influencing affect. Rumination is a repetitive thought process specific to depression, whereas worry is specific to anxiety (Watkins, 2008). Targeting mind wandering rather than rumination and worry offers two key advantages. First, by definition, mind wandering encompasses both rumination and worry (Robison et al., 2017). Therefore, exploring mind wandering enables the examination of broader issues beyond depression and anxiety. Second, rumination and worry have primarily been examined using subjective measures such as questionnaires (Meyer et al., 1990; Nolen-Hoeksema and Morrow, 1991). However, relying solely on questionnaires increases the risk of confounding factors such as respondents’ intentions during their responses or their self-monitoring abilities. Contrastingly, mind wandering can be measured using objective indicators such as cognitive tasks (Smallwood and Schooler, 2006). This overcomes the limitations of subjective measures and enables more empirical examination. The present study also employs subjective measures, but the replicability of the findings may be explored using objective measures.

Mind wandering has been shown to have positive aspects, such as promoting creativity (Mooneyham and Schooler, 2013). However, Yamaoka and Yukawa (2020) found that while inducing mind wandering enhances creativity, this increased creativity leads to heightened negative affect. Therefore, from a clinical psychology perspective, excessive mind wandering is a risk factor for poor mental health. Hence, the ability to control mind wandering may be beneficial. Moreover, various previous studies have attempted to distinguish between forms of mind wandering that are beneficial for mental health and forms that are detrimental. For example, mind-wandering episodes have been classified according to their intentionality (Seli et al., 2016), the presence or absence of meta-awareness (Seli et al., 2017a; Schubert et al., 2024), their temporal orientation (future- vs. past-oriented; Stawarczyk et al., 2013; Baird et al., 2011), and their emotional valence (positive vs. negative; Banks et al., 2016; Welz et al., 2018). These findings indicate that the emotional consequences of mind wandering depend not only on whether mind wandering occurs, but also on specific characteristics of its content, such as its emotional valence and temporal focus (e.g., Banks et al., 2016; Stawarczyk et al., 2013; Welz et al., 2018). However, these classifications are typically based on subjective self-report ratings. Given that mind wandering is fundamentally an attentional phenomenon, one potential advantage of studying mind wandering is that it can in principle be assessed using relatively objective performance-based indices. Furthermore, in many cognitive phenomena, more desirable and less desirable aspects tend to show substantial positive correlations (Diehl et al., 2024; Franklin et al., 2013; Soemer et al., 2024), suggesting that there may be limits to selectively enhancing only “good” forms of mind wandering while suppressing “bad” forms. Although attempts to distinguish “good” and “bad” mind wandering are undoubtedly informative, a consistently replicated finding is that a greater overall propensity for mind wandering is associated with poorer mental health (Fell, 2024; Webb et al., 2022). Therefore, rather than employing detailed subjective classifications of mind-wandering episodes as in some previous studies, the present study focuses on the general propensity for mind wandering.

Mindfulness training is effective in controlling mind wandering (Mrazek et al., 2012). Mindfulness is defined as the awareness gained by intentionally directing attention to the present moment without judgment (Kabat-Zinn, 2003), and mindfulness training is conducted to enhance state and/or dispositional mindfulness. Mindfulness training methods are diverse and include the raisin-eating exercise, body scans, mindfulness breathing, and stretching meditation (Segal et al., 2002). In mindful breathing, a representative method, participants continuously focus their attention on their spontaneous breathing; when their attention drifts away from their breathing, they return it to their breathing without evaluation or judgment, repeating this process. In the present study, we focus on dispositional (trait) mindfulness rather than state mindfulness. Dispositional mindfulness refers to a general tendency to attend to and be aware of present-moment experiences in daily life.

The mechanism by which mindfulness training controls mind wandering is thought to involve improvements in various cognitive functions, including executive functions (Mrazek et al., 2012; Mrazek et al., 2013). Mindfulness comprises two elements: self-regulation of attention and orientation toward experience (Bishop et al., 2004). Self-regulation of attention refers to observing and processing moment-to-moment changes in thoughts and sensations, whereas orientation to experience refers to maintaining an open and accepting attitude toward the present moment. Compassion is considered an important underlying factor in the latter orientation toward experience (Hofmann et al., 2011). Without compassion, one tends to make negative judgments about the object of attention, making it difficult to maintain state mindfulness. Therefore, compassion may be involved in the mechanism by which mindfulness training controls mind wandering in addition to cognitive functions. In recent years, self-compassion, which refers to compassion toward oneself, has been conceptually defined in psychology and has gained considerable attention. Self-compassion refers to the ability to maintain a balanced state of mind by holding compassionate feelings toward oneself when experiencing pain or worry, recognizing negative experiences as common to all humans, and balancing painful thoughts and affect (Neff, 2003).

Jazaieri et al. (2016) investigated the effects of mindfulness training aimed at improving compassion over 9 weeks on mind wandering using an experience sampling method. The results indicated that mindfulness training aimed at improving compassion reduced mind wandering. Greenberg et al. (2018) examined the effects of an 8-week mindfulness training program on self-compassion and mind wandering. The results indicated that an 8-week mindfulness training program improved self-compassion and reduced mind wandering; the moderating effect of self-compassion was significant, and when self-compassion improved through 8 weeks of mindfulness-based training, the effect of mind wandering on depression became insignificant. Cheung and Djekou (2024) also conducted a survey study with a sample of meditation practitioners. The results indicated that higher levels of mindfulness were associated with lower levels of mind wandering indirectly via higher levels of self-compassion. These findings suggest that self-compassion may be one process through which mindfulness is related to mind wandering. However, previous studies have not examined whether affect plays a mediating role in the associations among self-compassion, mindfulness, and mind wandering in nonclinical samples. Therefore, the present study focused on affect as a potential mediator linking self-compassion and dispositional mindfulness to mind wandering.

In cognitive science, the following background is assumed regarding the circumstances under which mind wandering occurs. Mind wandering is thought to occur during boring or familiar tasks and after difficult tasks, and increases over time. Therefore, it is considered to be a default state that arises when cognitive resources are depleted (Fortenbaugh et al., 2017; Randall et al., 2014; Thomson et al., 2015). In neuroscience, mind wandering has been shown to be associated with the activity of the default mode network, a grouping of interconnected brain regions that is active during rest (Kajimura et al., 2019). Furthermore, cognitive resources are the limited resources necessary for self-control, and once depleted, performance declines (Hagger et al., 2010). Thus, when cognitive function is high, it is possible to allocate the necessary cognitive resources to the task at hand (Gazzaley and Nobre, 2012; Sarter et al., 2001). However, when cognitive function is low, it is assumed that the allocation of cognitive resources fails, leading to mind wandering, which is the default state. Furthermore, because negative affect has been shown to deplete cognitive resources through control efforts (Beevers et al., 1999), it can be assumed that when negative affect arises, the cognitive resources that need to be allocated to the task at hand are depleted, resulting in wandering of the default state of mind. Furthermore, positive affect has been shown to restore cognitive resources (Tice et al., 2007). Thus, it can be assumed that when positive affect arises, the cognitive resources needed to allocate to the current task can be secured.

In mindfulness research, self-compassion plays a central role in improving affect. It is assumed that increased self-compassion enables people to accept negative affect as it is rather than avoid it, which, in turn, leads to improved affect (Feldman and Kuyken, 2011). In traditional mindfulness training based on Buddhist psychology, self-compassion is not explicitly addressed; it is assumed that self-compassion develops naturally as mindfulness is cultivated (Frostadottir and Dorjee, 2019; Grossman, 2010). Participation in long-term mindfulness training programs has been shown to improve self-compassion (Bergen-Cico et al., 2013; Rimes and Wingrove, 2011). Moreover, when self-compassion improves through mindfulness training, it is more effective in improving affect than when self-compassion does not improve (Baer et al., 2012; Kuyken et al., 2010; Van Dam et al., 2011). Furthermore, a meta-analysis has shown that self-compassion is effective in improving depression (*r* = −0.52), anxiety (*r* = −0.51), and stress (*r* = −0.54) (MacBeth and Gumley, 2012). Based on the above, improvements in affect through self-compassion may prevent the depletion of cognitive resources and help control mind wandering.

In research by Jazaieri et al. (2016) and Greenberg et al. (2018), no investigation was conducted assuming that self-compassion serves as a process controlling mind wandering, with affect as a mediating variable. Therefore, this study examined the relationship between self-compassion, affects, and mind wandering by comparing it with dispositional mindfulness. By examining these relationships, more detailed insights into the mechanisms by which mindfulness training controls mind wandering will be gained.

In the field of compassion research, it has recently been proposed that positive affect can be classified into two types: activated positive affect, which is associated with dopamine (e.g., “lively,” “excited”), and non-activated positive affect, which is associated with oxytocin (e.g., “safe,” “warm”; Gilbert et al., 2008). We return to this distinction when interpreting the present findings in the Discussion. In the present study, however, we measured positive affect using the Positive and Negative Affect Schedule, which is one of the most widely used instruments internationally and has been shown to be related to self-compassion (Krieger et al., 2015; López et al., 2015; Sujamani and Kanth, 2023).

*H1*: Mind wandering will negatively correlate with positive affect and positively correlate with negative affect.

*H2*: Self-compassion and dispositional mindfulness will be positively correlated with positive affect and negatively correlated with negative affect. Self-compassion will more strongly correlate with affect than dispositional mindfulness.

*H3*: Self-compassion will show a stronger negative association with mind wandering than dispositional mindfulness, through higher positive affect and lower negative affect.

## 2 Methods

### 2.1 Participants

A total of 176 undergraduate and graduate students from Japan participated in the survey. A total of 168 respondents with complete answers (62 men, 105 women, and one participant did not report sex; mean age 21.3 years, *SD* = 2.9) were included in the final analysis. The inclusion criteria were all undergraduate and graduate students who, after receiving an explanation of the study, provided informed consent to participate. On ethical grounds, individuals were excluded if they had current health concerns, such as receiving psychiatric treatment or counseling, or if they felt physically or mentally unwell at the time of completing the questionnaire.

### 2.2 Measures

(1) Japanese version of the Mind Wandering Questionnaire (Kajimura and Nomura, 2016; Mrazek et al., 2013): The MWQ is a five-item scale that assesses the general tendency to experience mind wandering. Participants responded on a 6-point Likert scale. Example items include “I have difficulty maintaining focus on simple or repetitive work” and “While reading, I find I have not been thinking about the text and must therefore read it again.” Cronbach’s alpha in the present sample was 0.74. Higher scores indicate a greater tendency toward everyday mind wandering.

(2) Six-factor mindfulness scale (Maekawa and Koshikawa, 2015): The SFMS is a measure of dispositional mindfulness. It consists of 31 items: 3 items for Nonduality, 3 items for Describing, 9 items for Acceptance and Nonreactivity, 3 items for Objective Observing, 7 items for Awareness, and 6 items for being in the Moment. In the present study, we used the total score as an index of overall dispositional mindfulness. Participants responded on a 5-point Likert scale. Maekawa and Koshikawa (2015) reported adequate reliability and validity for the SFMS in Japanese nonclinical samples. Example items include “I can observe my emotions objectively” and “I can accept my experiences without evaluating or judging them as good or bad”. Cronbach’s alpha for the total SFMS score in the present sample was 0.93. Higher scores indicate higher levels of dispositional mindfulness. The Five Facet Mindfulness Questionnaire (FFMQ; Baer et al., 2006) is one of the most widely used measures of dispositional mindfulness. However, it has been noted that some FFMQ items may be difficult to answer for individuals without prior mindfulness experience (Inoue, 2014). In addition, one of the FFMQ factors consists solely of reverse-scored items. Although reverse-scored items have the advantage of reducing acquiescent response bias, it has been suggested that factors composed mainly of such items may partly reflect response tendencies associated with reverse scoring (Maekawa, 2014). To address these issues, the SFMS was developed in Japan as a multifaceted mindfulness scale that retains a structure similar to that of the FFMQ while using straightforward, positively worded items that are easier for Japanese respondents to understand and endorse.

(3) Japanese version of the Self-Compassion Scale (Arimitsu, 2014; Neff, 2003): The SCS is a 26-item scale that assesses the tendency to be self-compassionate. Participants responded on a 5-point Likert scale. Example items include “I try to be understanding and patient toward those aspects of my personality I do not like” and “I’m kind to myself when I’m experiencing suffering.” Cronbach’s alpha for the total SCS score in the present sample was 0.91. Higher scores indicate a higher level of self-compassion.

(4) Japanese version of the Positive and Negative Affect Schedule (Sato and Yasuda, 2001; Watson et al., 1988): The PANAS is a scale that measures positive and negative affect, consisting of 8 items for Positive Affect and 8 items for Negative Affect. Participants responded on a 6-point Likert scale. In the present study, they were instructed to rate “how you generally feel in your everyday life.” Positive affect items include words such as “Active” and “Proud,” whereas negative affect items include words such as “Distressed” and “Ashamed.” Cronbach’s alpha was 0.87 for Positive Affect and 0.88 for Negative Affect. Higher scores indicate higher levels of typical positive or negative affect, respectively.

### 2.3 Procedure

We conducted a cross-sectional survey at a single time point using an anonymous web-based questionnaire. The survey was conducted using Google Forms. In several courses, after obtaining permission from each lecture, I posted the URL of the Google Form on the online learning management system that was accessible only to students enrolled in those courses. In addition, the first author asked undergraduate and graduate students who were acquaintances but were not informed of the details of the study plan to participate, and the URL of the Google Form was also distributed to them. The respondents were asked to complete the survey at their convenience using a computer, smartphone, or tablet.

Participants were informed that their responses would be anonymous and voluntary and would not affect their academic performance or course credits. Additionally, the participants were asked to refrain from participating if they were currently receiving medical treatment, taking medication, undergoing counseling, or feeling unwell. Research ethics were explained in the information section of the Google Form, and responses were only from those who agreed to participate. This study was approved by the Waseda University Academic Research Ethical Review Committee (Approval Number: 2020–106).

### 2.4 Statistical analyses

First, descriptive statistics for mind wandering, self-compassion, dispositional mindfulness, positive affect, and negative affect were calculated. Cronbach’s alpha coefficients were calculated to verify the reliability of each scale.

Second, Pearson’s product–moment correlation coefficients were calculated to examine the relationships among mind wandering, self-compassion, dispositional mindfulness, positive affect, and negative affect.

Third, to examine whether positive and negative affect statistically mediated the associations of self-compassion and dispositional mindfulness with mind wandering, we conducted a multiple mediation analysis with self-compassion or dispositional mindfulness as the explanatory variables, positive and negative affect as the mediating variables, and mind wandering as the dependent variable. The indirect associations between affect and mind wandering (often referred to as indirect effects in mediation analysis) were examined using 95% confidence intervals based on the percentile method with 2,000 bootstrap samples to assess the significance of the indirect paths (Preacher and Hayes, 2008).

All statistical analyses were conducted using R version 4.0.4 (R Foundation for Statistical Computing, Vienna, Austria).

## 3 Results

### 3.1 Descriptive statistics

Table 1 presents the results of the descriptive statistics and reliability coefficients for each scale. The reliability coefficients of each scale were generally sufficient.

**Table 1:** Descriptive statistics and correlations among study variables.

Variable	Mind wandering	Self-compassion	Dispositional mindfulness	Positive affect	Negative affect
1. Mind wandering	–				
2. Self-compassion	−0.16^*^	–			
3. Dispositional mindfulness	−0.18^*^	0.68^***^	–		
4. Positive affect	0.01	0.49^***^	0.56^***^	–	
5. Negative affect	0.30 ^***^	−0.55^***^	−0.42^***^	−0.11	–
Mean	18.17	17.84	95.05	27.07	24.01
Standard Deviation	4.52	3.84	19.63	7.97	8.65
Cronbach’s alpha coefficient	0.74	0.91	0.93	0.87	0.88

***p < 0.001, *p < 0.05.

### 3.2 Correlation analysis

The results of the correlation analysis showed that self-compassion was significantly and positively correlated with dispositional mindfulness (*r* = 0.68, *p* < 0.001). The correlation between positive affect and negative affect was not significant (*r* = −0.11, *p* = 0.17). Regarding the relationship between mind wandering and affect, mind wandering showed a significant positive correlation with negative affect (*r* = 0.30, *p* < 0.001) but no significant correlation with positive affect (*r* = 0.01, *p* = 0.94). Regarding the relationship between self-compassion and affect, self-compassion was positively correlated with positive affect (*r* = 0.49, *p* < 0.001) and negatively correlated with negative affect (*r* = − 0.55, *p* < 0.001). Regarding the relationship between dispositional mindfulness and affect, dispositional mindfulness showed a positive correlation with positive affect (*r* = 0.56, *p* < 0.001) and a negative correlation with negative affect (*r* = − 0.42, *p* < 0.001). Additionally, mind wandering showed a significant negative correlation with self-compassion (*r* = −0.16, *p* = 0.04) and dispositional mindfulness (*r* = −0.18, *p* = 0.02; Table 1).

### 3.3 Multiple mediation analysis

**Figure 1:**
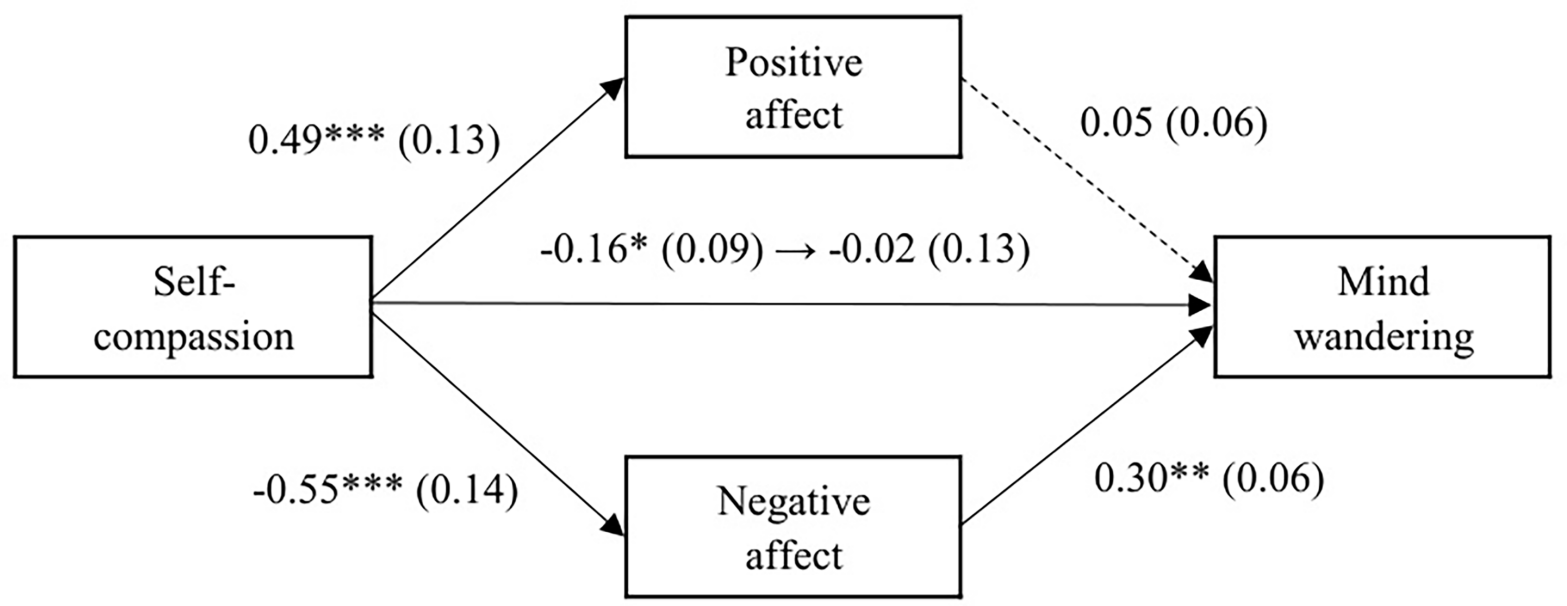
Results of the multiple mediation analysis with self-compassion as the predictor. Note. Standardized coefficients are shown, with standard errors in parentheses. ***p < 0.001, **p < 0.01, *p < 0.05.

The results of the multiple mediation analysis with self-compassion as the explanatory variable are shown in Figure 1. The overall association between self-compassion and mind wandering was significant, indicating that higher self-compassion was associated with lower levels of mind wandering (*β* = −0.16, SE = 0.09, *p* = 0.04). However, when the mediating variable was controlled for, the direct effect became non-significant (*β* = −0.02, SE = 0.13, *p* = 0.88). Self-compassion was positively associated with positive affect (*β* = 0.49, SE = 0.13, *p* < 0.001), and negatively associated with negative affect (*β* = −0.55, SE = 0.14, *p* < 0.001). When one of the mediating variables was controlled for, negative affect was positively associated with mind wandering (*β* = 0.30, SE = 0.06, *p* = 0.01), but the effect of positive affect on mind wandering was not significant (*β* = 0.05, SE = 0.06, *p* = 0.65). The results of the indirect effect analysis using the bootstrap method showed that the overall indirect effect was not significant (*β* = −0.14, SE = 0.11, 95% CI [−0.39, 0.05]). Next, we examine the indirect effects of each mediating variable. The indirect effect of positive affect was not significant (*β* = 0.02, SE = 0.06, 95% CI [−0.09, 0.16]), but the indirect effect of negative affect was significant (*β* = −0.16, SE = 0.08, 95% CI [−0.37, −0.06]), indicating that self-compassion was indirectly associated with lower mind wandering through lower negative affect.

**Figure 2:**
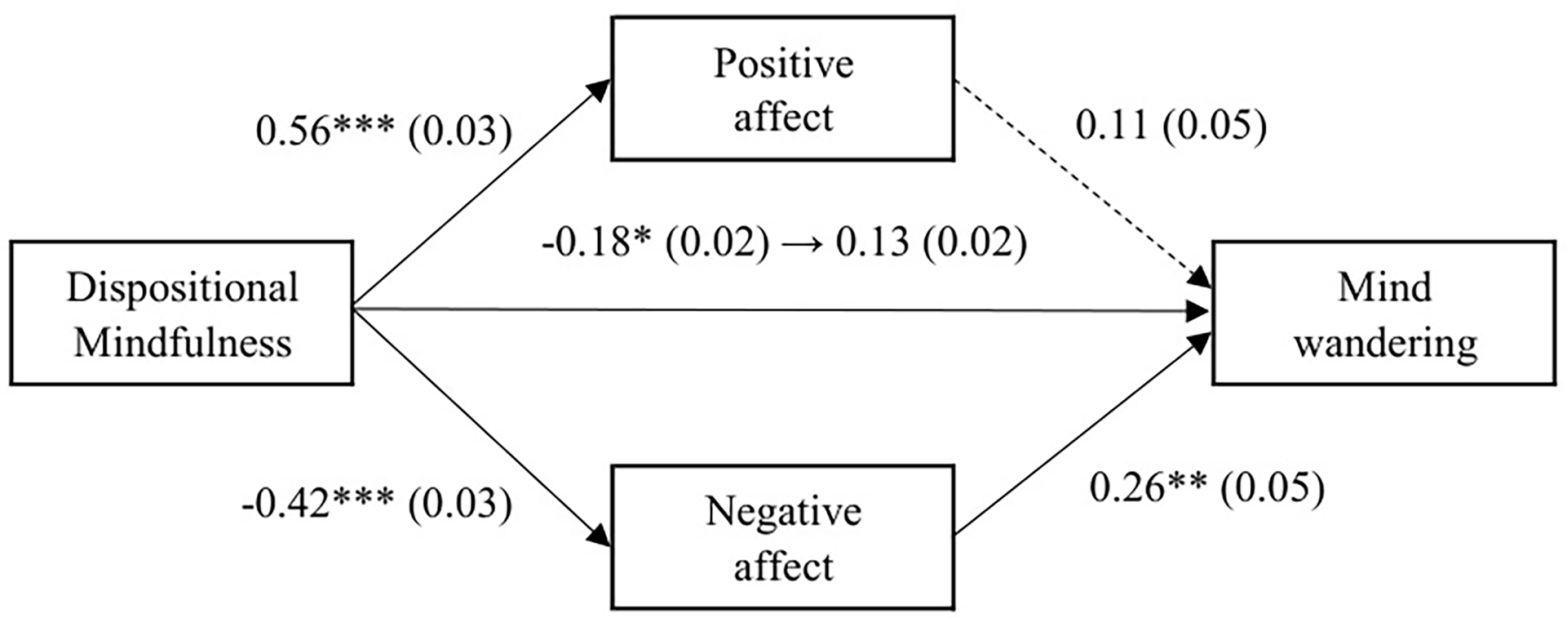
Results of the multiple mediation analysis with dispositional mindfulness as the predictor. Note. Standardized coefficients are shown, with standard errors in parentheses. ***p < 0.001, **p < 0.01, *p < 0.05.

The results of the multiple mediation analysis with dispositional mindfulness as the explanatory variable are shown in Figure 2. The overall association between dispositional mindfulness and mind wandering was significant, indicating that higher dispositional mindfulness was associated with lower levels of mind wandering (*β* = −0.18, SE = 0.02, *p* = 0.03). However, when the mediating variable was controlled for, the direct effect became non-significant (*β* = −0.13, SE = 0.02, *p* = 0.16). Dispositional mindfulness was positively associated with positive affect (*β* = 0.56, SE = 0.03, *p* < 0.001), and negatively associated with negative affect (*β* = −0.42, SE = 0.03, *p* < 0.001). When one of the mediating variables was controlled for, negative affect was positively associated with mind wandering (*β* = 0.26, SE = 0.05, *p* < 0.001), but the effect of positive affect on mind wandering was not significant (*β* = 0.11, SE = 0.05, *p* = 0.27). The results of examining the indirect effects using the bootstrap method showed that the overall indirect effect was not significant (*β* = −0.05, SE = 0.02, 95% CI [−0.04, 0.02]). Next, the results of examining the indirect effects of each mediating variable showed that the indirect effect of positive affect was not significant (*β* = 0.06, SE = 0.01, 95% CI [−0.01, 0.04]), the indirect effect of negative affect was significant (*β* = −0.11, SE = 0.01, 95% CI [−0.05, −0.01]), indicating that dispositional mindfulness was indirectly associated with lower mind wandering through lower negative affect.

## 4 Discussion

This study aimed to examine how self-compassion is associated with affect and the control of mind wandering, in comparison with dispositional mindfulness.

The results of the correlation analysis revealed a significant positive correlation between self-compassion and dispositional mindfulness, supporting the findings of previous studies (Bergen-Cico et al., 2013; Rimes and Wingrove, 2011).

Regarding the relationship between mind wandering and affect, mind wandering was found to be positively correlated with negative affect, supporting previous studies (e.g., Killingsworth and Gilbert, 2010; Mrazek et al., 2013; Poerio et al., 2013) showing that mind wandering is associated with worsening affect. However, mind wandering was not related to positive affect. Therefore, H1 was supported concerning negative affect but not positive affect.

Furthermore, regarding the relationship between self-compassion, dispositional mindfulness, and affect, the results of the correlation analysis showed that self-compassion and dispositional mindfulness were positively correlated with positive affect and negatively correlated with negative affect. The correlation coefficient was higher for dispositional mindfulness than for self-compassion concerning positive affect (self-compassion: *r* = 0.49; dispositional mindfulness: *r* = 0.56), while the correlation coefficient was higher for self-compassion than for dispositional mindfulness about negative affect (self-compassion: *r* = − 0.55; dispositional mindfulness: *r* = − 0.42). Therefore, H2 was supported for negative affect but not for positive affect. Furthermore, self-compassion and dispositional mindfulness were negatively correlated with mind wandering.

The multiple mediation analysis indicated that the total indirect effect was not significant in either model, whether self-compassion and dispositional mindfulness was used as the explanatory variable. Thus, both models examined in this study were insufficient in fully explaining the relationships among the variables. Consequently, the discussion focuses primarily on the significant findings. The indirect path via negative affect was significant, suggesting that self-compassion and dispositional mindfulness may be related to lower levels of mind wandering through lower negative affect. Notably, self-compassion showed a larger absolute *β* value than dispositional mindfulness in its association with negative affect (self-compassion: *β* = −0.55; dispositional mindfulness: *β* = −0.42). Moreover, self-compassion and dispositional mindfulness were positively associated with positive affect, but the indirect association of positive affect with mind wandering was not significant. Therefore, H3 is supported for negative affect but not for positive affect.

These results suggest that self-compassion shows a stronger negative association with negative affect than dispositional mindfulness and may be particularly relevant to understanding how negative affect and everyday mind wandering are related. This pattern is consistent with meta-analytic evidence showing that higher self-compassion is strongly associated with lower depression, anxiety, and stress (MacBeth and Gumley, 2012). However, as we did not conduct tests directly comparing self-compassion and dispositional mindfulness, the results should be interpreted with caution. While the findings of this study are suggestive, they are consistent with prior research (Baer et al., 2012; Kuyken et al., 2010; Van Dam et al., 2011) indicating that increases in self-compassion are associated with greater improvements in affect during mindfulness training. High self-compassion, as shown in prior research, promotes active acceptance of negative affect rather than avoidance (Leary et al., 2007). Therefore, higher levels of self-compassion may be associated with lower negative affect without having a paradoxical effect on thought suppression.

Furthermore, reducing negative affect may be more important than increasing positive affect for the control of mind wandering, given that negative affects have been shown to increase mind wandering and to shape its content (Smallwood et al., 2009; Stawarczyk et al., 2013). Consistent with previous findings (MacBeth and Gumley, 2012), our results also showed that lower self-compassion was associated with higher negative affect. Given that negative affect depletes cognitive resources (Beevers et al., 1999), it can be assumed that there are insufficient cognitive resources to allocate to the task at hand, resulting in mind wandering, which is considered the brain’s default state in cognitive neuroscience. Although positive affect has been shown to restore cognitive resources (Tice et al., 2007), the present findings tentatively suggest that, for the control of mind wandering, preventing the depletion of cognitive resources by negative affect may be more important than restoring resources through positive affect.

As noted in the Introduction, recent research on compassion has classified positive affect into two types: active positive affect (e.g., “lively” and “excited”) associated with dopamine, and soothing positive affect (e.g., “safe” and “warm”) associated with oxytocin (Gilbert et al., 2008). Increased self-compassion has been shown to increase soothing positive affect (Petrocchi et al., 2017). The positive affect measured in the PANAS in this study can be interpreted as active positive affect. Therefore, different results could be obtained by changing the scale used. Positive affect is associated with the parasympathetic nervous system, which is the counterpart to negative affect (Gilbert et al., 2008; Petrocchi et al., 2017). Therefore, although the positive affect measured by the PANAS did not correlate with mind wandering, soothing positive affect may be related to it. However, because mind wandering is considered the default state of the brain in cognitive neuroscience (Randall et al., 2014; Kajimura et al., 2019), when soothing positive affect is activated and a person feels relaxed and secure, mind wandering may increase.

When soothing positive affect is high and a person feels relaxed and secure, mind wandering may increase. However, consistent with daily-life research showing that mind-wandering episodes with more pleasant content are associated with better subsequent affect (Welz et al., 2018), such mind wandering may not be the kind of mind wandering that leads to affect deterioration or mental illness. Rather, it might differ from ruminative, negatively valenced mind wandering that has been linked to poorer mental health (e.g., Hoffmann et al., 2016; Shin et al., 2015). By examining the relationship with non-activated positive affect in more detail, it may be possible to gain insights into mind wandering that does not lead to affect deterioration or mental illness.

The mechanisms by which mindfulness training reduces mind wandering have been examined primarily by focusing on improvements in executive function (Mrazek et al., 2012; Mrazek et al., 2013). This is because mind wandering is considered a phenomenon of attention (Smallwood and Schooler, 2006). Previous studies have shown that increased self-compassion reduces mind wandering; however, the mechanisms underlying this relationship have not been empirically examined (Greenberg et al., 2018; Jazaieri et al., 2016). Therefore, in this study, we examined the background of mind wandering and focused on cognitive resources rather than executive function. We then examined whether affect, which affects cognitive resources, influences the control of mind wandering. Furthermore, we examined and compared dispositional mindfulness and self-compassion. The results showed that dispositional mindfulness and self-compassion may be associated with lower levels of mind wandering through lower negative affect. This is in line with previous work indicating that self-compassion is strongly related to lower emotional symptoms (MacBeth and Gumley, 2012) and can buffer the link between mind wandering and depression (Greenberg et al., 2018). They also suggest that self-compassion may play a relatively stronger role than dispositional mindfulness in its association with negative affect.

This study suggests that the mechanisms linking dispositional mindfulness and mindfulness training to lower levels of mind wandering are not limited to improvements in executive function. Dispositional mindfulness and self-compassion may have the potential to alleviate the negative spiral between negative affect and mind wandering through their associations with lower negative affect. This idea is compatible with intervention studies showing that both mindfulness-based and compassion-focused programs can reduce emotional symptoms and rumination (Hofmann et al., 2011; Frostadottir and Dorjee, 2019). If there are individuals who are unable to practice mindfulness training or find it difficult to engage effectively, it may be useful to suggest ways to cultivate self-compassion or to support more adaptive regulation of affect through other methods.

The results of this study should be interpreted with caution. Although certain components of the mediation model demonstrated significant indirect paths and were discussed in detail, the total indirect effect was not statistically significant. Thus, the multiple mediation model as a whole was not supported, and the present findings should be considered preliminary. Future research is warranted to refine the proposed model, particularly with regard to the role of positive affect, and to further investigate potential causal mechanisms underlying these relationships. Second, no direct comparison between self-compassion and dispositional mindfulness was conducted. The finding that self-compassion may play a somewhat stronger role than dispositional mindfulness in its association with negative affect should be interpreted as suggestive rather than conclusive. Given the high correlation between mindfulness and self-compassion, more rigorous comparative analyses through experimental studies specifically designed to enhance both variables are necessary. Third, the effect sizes were small. Although some indirect associations in the mediation models reached statistical significance, none were large. Other unexamined factors may have influenced the results, warranting caution in their interpretation.

This study suggests two recommendations for future research. First, we did not examine each subscale of dispositional mindfulness and self-compassion separately. Specifically, research indicates that acceptance plays a crucial role in the effectiveness of mindfulness for emotion regulation (Lindsay and Creswell, 2019). However, mindfulness is not solely comprised of acceptance, and all components are interrelated. To avoid overcomplicating the model, this study did not examine each subscale. Nevertheless, examining each subscale in future studies may facilitate a more detailed investigation of the control mechanisms for mind wandering. Second, the relationships between the various factors require further examination. This study suggests that dispositional mindfulness and self-compassion may help to mitigate the negative cycle between negative affect and mind wandering through their associations with lower negative affect. However, the influence of negative affect on mind wandering was not examined. Further investigation into how dispositional mindfulness, self-compassion, mind wandering, and emotions influence each other is recommended.

### 4.1 Limitations

This study has several limitations. First, all variables were assessed using self-report questionnaires. Although mind wandering is fundamentally an attentional phenomenon that can, in principle, be indexed using performance-based measures, we did not include such objective indicators; therefore, it remains unclear whether similar patterns would be observed with behavioral or physiological measures. Furthermore, we assessed dispositional mindfulness using the Six-Factor Mindfulness Scale (SFMS), which was developed in Japan. As no validated versions are currently available in other languages, it is uncertain whether the SFMS would show adequate reliability and validity when translated or applied in different cultural contexts. Replication with other measures and in other cultural settings is needed. Moreover, we focused on dispositional mindfulness and did not assess state changes in mindfulness during specific practices. Future studies could examine whether state increases in mindfulness show similar associations with affect and mind wandering.

Second, the cross-sectional design precludes causal inferences about the relationships among self-compassion, dispositional mindfulness, affect, and mind wandering. Longitudinal and intervention studies are required to test whether increases in mindfulness and self-compassion through training lead to changes in affect and mind wandering.

Third, our sample consisted of non-clinical Japanese university and graduate students. We did not assess additional individual-difference variables such as depressive and anxiety symptoms, ADHD-related traits, or trait rumination, nor did we systematically assess participants’ prior experience with mindfulness or self-compassion practices. As meditation experts or individuals with higher levels of psychological difficulties may show different patterns, the generalizability of our findings to more diverse or clinical populations is limited. Future research should examine whether the observed associations hold across different age groups, cultures, and clinical statuses.

Fourth, in line with our focus on the general propensity for mind wandering, we used a global self-report measure of mind wandering and did not assess the detailed content or type of mind-wandering episodes (e.g., positive vs. negative, future- vs. past-oriented, intentional vs. unintentional), nor did we distinguish between potentially “adaptive” and “maladaptive” forms of mind wandering. Recent work suggests that the impact of mind wandering on affect depends on such characteristics. Consequently, we could not determine whether the present associations are driven primarily by particular types of mind wandering. Future studies should combine global measures of mind-wandering propensity with more fine-grained assessments of mind-wandering content and type.

## Data Availability

The raw data supporting the conclusions of this article will be made available by the authors, without undue reservation.
